# Comparative transcriptome analysis of longissimus dorsi muscle reveal potential genes affecting meat trait in Chinese indigenous Xiang pig

**DOI:** 10.1038/s41598-024-58971-2

**Published:** 2024-04-11

**Authors:** Wei Wang, Dan Wang, Xinyi Zhang, Xiaoli Liu, Xi Niu, Sheng Li, Shihui Huang, Xueqin Ran, Jiafu Wang

**Affiliations:** Institute of Agro-Bioengineering/Key Laboratory of Plant Resource Conservative and Germplasm Innovation in Mountainous Region and Key Laboratory of Animal Genetics, Breeding and Reproduction in the Plateau Mountainous Region (Ministry of Education), College of Life Science and College of Animal Science, Guizhou University, Guiyang, 550025 China

**Keywords:** Biochemistry, Computational biology and bioinformatics, Molecular biology

## Abstract

In this study, we compared the transcriptome of longissimus dorsi muscle between Guizhou Xiang pigs (XP) and Western commercial Large White pigs (LW), which show diffirent meat quality between them. In terms of meat quality traits, the pH 45 min, color score, backfat thickness, and intramuscular fat (IMF) content were higher in Xiang pigs than in Large White pigs (P < 0.01), while the drip loss, lean meat percentage, shear force, and longissimus dorsi muscle area of Xiang pigs were lower than that of Large White pigs (P < 0.01). Nutrients such as monounsaturated fatty acid (MUFA), total amino acids (TAA), delicious amino acids (DAA) and essential amino acids (EAA) in Xiang pigs were higher than that in Large White pigs, and the proportion of polyunsaturated fatty acid (PUFA) of Xiang pigs was significantly lower than Large White pigs (P < 0.01). Transcriptome analysis identified 163 up-regulated genes and 88 genes down-regulated in Xiang pigs longissimus dorsi muscle. Combined with the correlation analysis and quantitative trait locis (QTLs) affecting meat quality, a total of 227 DEGs were screened to be significantly associated with meat quality values. Enrichment analysis indicated that numerous members of genes were gathered in muscle development, adipogenesis, amino acid metabolism, fatty acid metabolism and synthesis. Of those, 29 genes were identified to be hub genes that might be related with the meat quality of Xiang pig, such as *MYOD1, ACTB, ASNS, FOXO1, ARG2, SLC2A4, PLIN2,* and *SCD.* Thus, we screened and identified the potential functional genes for the formation of meat quality in Xiang pigs, which provides a corresponding theoretical basis for the study of the molecular regulatory mechanism of pork quality and the improvement of pork quality.

## Introduction

Pork plays a major role in meat consumption, and is the most produced and consumed meat in the world. With the improving of the standard of living, people are pursuing high-quality meat to tackle the sustainability challenges of meat production and consumption^1^. Meat quality is not only an important economic trait for the pork industry, and it also has a direct impact on human health and the life^2,3^. Meat quality is a comprehensive concept that is difficult to measure in a simple and unique way, the indicators used to evaluate meat quality and as the basis for pork selection by consumers, including the meat color, marbling, pH, tenderness, shear force, water loss^4^. The intramuscular fat (IMF) consists of phospholipids, triglycerides, and cholesterol, and the level of IMF is reflected in the balance between the synthesis, degradation, and absorption of triglycerides. Intramuscular triglycerides are mainly stored in adipocytes and also in the cytoplasm of muscle fibers in the form of droplets^5^. The IMF content is highly correlated with the sensory acceptability of pork, water holding capacity and tenderness. When the IMF content is higher than 2.5%, the flavor and juiciness of pork are enhanced^6–8^. The IMF deposition and muscle fiber type are important for assessing the potential eating quality of pork loins, which are correlated with particular flavour, juiciness and tenderness^9,10^. Nutrient composition is also an important part of meat quality. As a precursor of fat, fatty acid is one of a major factor affecting the tenderness and flavor of meat. In addition, a number of amino acids have a large impact on the flavor, such as glutamate, aspartic, leucine, and lysine, some of which are good indicator of the protein content of meat^11^.

Meat quality traits are influenced by a variety of factors, such as the environment, dietary management, and especially, genetics^12^. Previous reports have focused on quantitative trait loci (QTL) and intrinsic genes associated with meat quality and explored the relationship between genes and meat quality at the molecular level, and many genes and QTLs have been identified to have a linkage with meat quality in pigs^13–15^. In addition, a series of genes related to the meat quality of pigs have been identified, such as acetyl-CoA carboxylase alpha (*ACACA)*, ELOVL fatty acid elongase 5 (*ELOVL5)*, succinate-CoA ligase GDP-forming subunit beta (*SUCLG2)*, isocitrate dehydrogenase (NADP(+)) 2 (*IDH2)*, and myogenic differentiation 1 (*MYOD1)*^16–18^. With the advantages of high feed conversion efficiency, fast growth rate and obvious economic benefits, the European pig breeds have the largest market share in China and even in the world, but it has the defect of producing poor quality pork, which has caused consumer dissatisfaction^19^. Local Chinese native pig breeds are usually well adapted to specific environmental conditions and feed resources, and are considered to have better meat quality and are able to produce high quality meat products compared to western pig breeds^20,21^. Several studies have been conducted to detect differentially expressed genes (DEGs) associated with meat quality using transcriptomic information from the longissimus dorsi muscle and adipose tissue of Western commercial pig breeds and Chinese local pig breeds^16,22,23^. Nevertheless, previous works aims at several Chinese endemic pig breeds, accurate information on the degree of difference in meat quality traits is still limited, and it is not possible to clearly illustrate the differential diversity of meat quality in Eurasian pig breeds.

Xiang pig is a miniature and invaluable Chinese indigenous pig breed that originated in the southeastern regions of Guizhou province, China, and particularly, Xiang pig has been well-known for its higher IMF content, tender texture, delicious taste, and rich flavor^24,25^. However, the IMF content and meat quality in Xiang pigs and European pigs varied considerably, and the differential mechanism is still unclear. Hence, it can serve as an appropriate animal model for studying of the IMF deposition and regulatory mechanism underlying the formation of superior meat quality traits. In the present study, we investigate the histological structure of longissimus dorsi muscle, and compared the meat quality indicators, fatty acid content and amino acid content in the longissimus dorsi muscle of Xiang pigs and Large White pig breeds. To explore the molecular mechanisms and screen genes related with meat quality, the longissimus dorsi muscle were sampled from Xiang pigs and Large White pigs, respectively. We applied RNA-seq to investigate the expression patterns of mRNA in longissimus dorsi muscle tissue of Xiang pigs and Large White pigs. Our findings will provide new insights into the molecular mechanisms of IMF deposition and the formation of good meaty traits, which may provide a foundation for the production of high-quality pork.

## Materials and methods

### Ethics statement and animal sample collection

All animal procedures were approved by the guidelines of Guizhou University Subcommittee of Experimental Animal Ethics with no. of EAE-GZU-2020-P002. Also, all the experiments in the manuscript follows the recommendations in the Animal Research Reporting in Vivo Experiments (ARRIVE) guidelines.

Ten Xiang pigs and ten Large White pigs were randomly collected from Guizhou Dashandi Ecological Breeding Co., Ltd, China. All animals selected were male with an mean age of 5 months and 15 days. The detail of the age and gender of the animals were shown in Table S1. All selected pigs were raised under the same temperature, humidity, ventilation conditions and feeding standards, and animals received the same diet all their life. The composition of the animal feed was shown in Table S2. The animals were euthanized by electric shock and rapid bleeding, and all collected longissimus dorsi muscle samples were measured for meat quality indexes and frozen in liquid nitrogen immediately and stored at − 80 °C until subsequent experiments were performed.

### Histological examination

The longissimus dorsi muscle of Xiang pigs and Large White pigs were quickly fixed in 4% paraformaldehyde. Then, the wax blocks were dehydrated and embedded, the slices were cut to a thickness of 5 µm using a paraffin microtome, eluted with epoxy resin and stained with hematoxylin and eosin (H&E). Subsequently, images of the cross-sections stained with H&E were captured by Microtome Scanner (Pannoramic MIDI, 3DHISTECH, Hungary), and three images of scale bar = 50 μm were selected for analysis of myofiber density and diameter using ImageJ software, all muscle fibers in the same field of view were analyzed.

### Meat quality assessment

The pH_45_ value of the longissimus dorsi muscle was measured by a hand-held pH meter (pH-STAR, SFK-Technology, Denmark) at 45 min after slaughter. The drip loss was determined using longissimus dorsi muscle cut into 2 cm × 3 cm × 2 cm sizes, suspended in 4 °C for 24 h, and weighed again, (Drip loss percentage = (initial weight − final weight)/initial weight × 100%). Color measurement was performed within 1 h after slaughter, and standard meat colorimetric cards (NPPC) were used for meat color determination. The backfat was taken from the last rib, the backfat thickness was measured with a straightedge. Lean meat percentage was calculated after slaughter (Lean meat percentage (%) = Lean meat weight / (skin weight + fat weight + muscle weight + bone weight) × 100). Shear force was directly determined by the C-LM3B digital display muscle tenderness meter (Tenovo International Co., Limited. Beijing, China). Longissimus dorsi muscle area at the last rib was traced on sulphate papers and calculated it by ImageJ software. Each sample was measured 3 times and the mean value was used for further analysis.

### Fatty acid and amino acid content analysis

The IMF content was measured by the Soxhlet extraction method^26^. Briefly, all longissimus dorsi muscle samples were dried to constant weight in the oven and then grinded into powder, extracted with petroleum ether solution to obtain IMF. Extracted lipid was added 2 mol/l KOH in methanol: water (1:1 v/v), and 2 mL of n-hexane for saponification and methylation. Then, fatty acid methyl esters were processed by gas chromatograph Trace1310 ISQ (Thermo Scientific, USA, Column: TraceGOLD™ TG-WaxMS A GC) for Xiang pigs and Large White pigs. All fatty acid standards were from Shanghai Amperexperiment Technology Co, China. The signal for each fatty acid was quantified as a percentage of the total fatty acid methyl ester.

The amino acid content in longissimus dorsi muscle was analyzed by Liquid Chromatograph (1260, Agilent, USA). Briefly, about 0.2 g of freeze-dried muscle sample powder was hydrolyzed in a sealed ampoule bottle with 10 mL of hydrochloric acid solution (HCl, 6 mol/L) for 24 h at 110 (± 1) °C. All the hydrolysis products were diluted to 25 mL by 0.02 mol/L hydrochloric acid solution, and 100 μL of sample was added to a 15 mL centrifuge tube and dried in a vacuum oven at 60 °C for 2 h. The centrifuge tube was filled with nitrogen and added 50 μL of derivatization reagent (ethanol:phenyl isothiocyanate:water:triethylamine = 7:1:1:1), derivatization was carried out for 30 min at room temperature, and a mixture of anhydrous sodium acetate and acetonitrile was added to 0.5 mL. Agilent liquid chromatography (Column: C18 SHISEIDO (4.6 mm*250 mm*5 μm), Wavelength: 254 nm) was used for amino acid analysis. All amino acid standards were from Shanghai Amperexperiment Technology Co, China, and the amino acid content of the samples was quantified by comparing the peak profiles of the obtained samples with standard amino acid spectra.

### Library construction and sequencing

Five biological replicates of each breeds were used for sequencing. Total RNAs of the longissimus dorsi muscle tissue were isolated individually with Trizol reagent (Tiangen Biotech, Beijing, China). The quantity and integrity of the total RNA was assessed by electrophoresis on a 1% agarose gel and using the Agilent 2100 Size Bio-analyzer system (Agilent Technologies, CA, USA). The cDNAs were synthesized for RT-qPCR using the PrimeScript™ II 1st Strand cDNASynthesis Kit (TaKaRa Bio, China). Approximately 5 μg of total RNAs per sample was used for sequencing library preparation using a NEBNext ® Ultra ™ Directional RNA Library Prep Kit for Illumina ® (NEB, Ipswich, MA, USA). The ribosomal RNAs were removed using a Ribo-Zero ™ GoldKits (Epicentre, Madison, WI, USA). The RNAs were fragmented and reverse transcribed using the TruSeq RNA LT/HT Sample Preparation Kit (Illumina, USA). The library preparations were sequenced on an Illumina HiSeqTM 2000 sequencing platform after the quality of the cDNA libraries were qualified by Bioanalyzer 2200 evaluation (Agilent, Santa Clara, CA), and generated 100 bp paired-end reads according to the manufacturer's protocols at the Beijing Genomies Institute (BGI), Shenzhen, China.

### Dataset analysis

The sequencing data from ten libraries were taken for the analysis of the mRNA expression profile by RNA-seq method. Data analysis included initial quality control and filtering steps. Raw reads in fastq format from each sequencing library were filtered to remove low quality reads by Trimmomatic (version0.39) with default parameters. Then, clean reads were mapped to the pig reference genome (Sscrofa 11.1) using HISAT2 (version 2.1.0) with default parameters. The mapped sam files were sorted and converted into bam files by SAMtools (version 0.1.19). In order to obtain the mRNA abundance of all genes and construct the gene expression profile in the longissimus dorsi muscle, Subread featurecounts (version2.0.0) was used to count the reads amount. CPM values (counts per million mapped reads, CPM = mRNA read counts ÷ total mapped read counts × 10^6^) were calculated by formula to estimate the expression level of genes, in which all of CPM values were added 0.001 for logarithm arithmetic. The minimum normalized CPM was 1.0, in which a gene would be eliminated if its CPM value of any sample was not lager than the threshold, which can eliminate the large library differences between samples caused by small differences.

Based on the CPM values, edgeR were used to analyze the difference in gene expression. The threshold for DEGs was |log2 (fold_change)|> 0.95, P < 0.05 and FDR < 0.05. Then the Ensembl IDs were converted into the gene names and biological function annotation of pig via BioMart online (http://www.ensembl.org/biomart). Gene Ontology (GO) functional annotations and Kyoto Encyclopedia of Genes and Genomes (KEGG) pathway enrichment analysis of DEGs were performed with the KOBAS (http://bioinfo.org/kobas/genelist/) and Omic Share (https://www.omicshare.com/tools) (Based on ORA), and reference Sscrofa11.1 as background based on Ontology Consortium (http://geneontology.org/) and KEGG for enrichment categories. Pathways and GO functional annotations (P < 0.01) that were statistically significantly enriched in DEGs were further explored. Pearson correlation between the expression of DEGs and meat quality indicators values were analyzed using R package Hmisc (https://cran.r-project.org/web/packages/Hmisc/index.html) in the data set measured in this study. To identify DEGs associated with porcine meat quality, the DEGs were compared with QTLs affecting meat quality collected in the pig QTL database (http://www.animalgenome.org/cgi-bin/QTLdb/SS/index, Release 50, Apr 25, 2023). QTLs with the same physical location were eliminated, and associated with meat quality were considered as usable QTL. We performed QTLs analysis using BEDTools (version 2.17.0). Protein Interaction Analysis (PPI) was performed using Search Tool for Retrieval of Interacting Genes (STRING).

### Validation of DEGs

To verify the reliability of the RNA-seq data, RT-qPCR was performed on 6 candidate genes (*ACTA1, ACADL, FOXO1, PFKFB3, PPARGC1A, SRPK3*). Five samples in each group were used as the same aliquot of total RNA for RNA-seq detection. Primers were designed by Primer 5.0, and the PCR condition and proportion were the same as our previous work with each primer concentration of 10 pmol/μL, the *GAPDH* gene and β-actin gene were used as internal controls^27^. Based on dissociation curve analysis for PCR products, the amplification efficiency was controlled within range of 100 ± 10%. The relative expression level of target gene utilized the method of 2(− ΔΔCt) as reported by Livak et al^28^. The different level of gene expression between two groups was tested by software GraphPad Prism (v8.0.2) taking the P < 0.05 as threshold of significant difference. The results were presented as mean ± standard deviation. The nucleotide sequences of primers were listed in Table S3.

### Statistical analysis

All data were analyzed by one-way ANOVAs using GraphPad Prism (v8.0.2) statistical software. Results were presented as means ± SD. Differences between means were considered statistically significant at P < 0.05.

## Results

### Comparison of histological structure and meat quality indicators in longissimus dorsi muscle of two breeds

**Figure 1:**
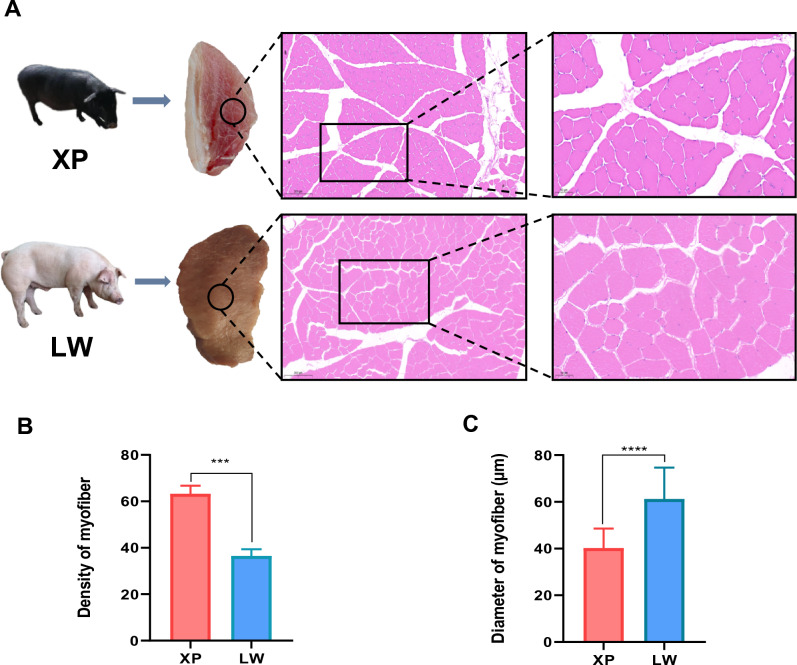
Histological observation of longissimus dorsi muscle. (**A**) HE staining of Xiang pigs and Large White pigs sections. Scale bar: 200 μm and 50 μm. (**B**) The density of myofiber of Xiang pigs and Large White pigs groups in the same field of view. (**C**) Diameter of myofiber of Xiang pigs and Large White pigs groups.

Compared with the Large White pigs, the Xiang pigs had significant marbling and a redder meat color in longissimus dorsi muscle. In addition, the backfat of the Xiang pig was thicker than that of the Large White pig (Fig. S1). The haematox-ylin-eosin (H&E) staining was used to evaluate the anatomical and histological structure of longissimus dorsi muscle. It was found that the muscle fibers of Xiang pigs were arranged in an oval shape, while those of Large White pigs were irregularly square (Fig. 1A). Our statistical analysis showed that the density of muscle fibers in Xiang pigs was higher than that in Large White pigs (Fig. 1B), the muscle fiber diameter of Xiang pigs was significantly lower than that of Large White pigs (Fig. 1C).

The meat quality indicators in longissimus dorsi muscle of Xiang pigs and Large White pigs were measured (Table 1). We found that pH 45 min (6.87 ± 0.24 vs 6.27 ± 0.23), color score (3.60 ± 0.39 vs 2.89 ± 0.65), backfat thickness (2.49 ± 0.73 vs 2.12 ± 0.61), and IMF content (5.65 ± 0.78 vs 2.43 ± 0.66) of Xiang pigs were higher than that of Large White pigs, while the drip loss (2.56 ± 1.02 vs 3.94 ± 0.97), lean meat percentage (45.64 ± 4.64 vs 63.29 ± 1.97), shear force (23.60 ± 2.16 vs 34.58 ± 6.30), and longissimus dorsi muscle area (15.89 ± 1.85 vs 58.52 ± 8.77) of Xiang pigs were lower than that of Large White pigs. These data demonstrated that the Xiang pigs group had a different meat quality compared with the Large White pigs group.

**Table 1:** Comparison of meat quality indicators of Xiang pigs and Large White pigs.

Traits	Xiang pigs (n = 10)	Large White pigs (n = 10)	Level of significance
	Mean ± SD	Mean ± SD	
pH 45 min	6.87 ± 0.24	6.27 ± 0.23	**
Color Score	3.6 ± 0.39	2.89 ± 0.65	**
Drip loss (%)	2.56 ± 1.02	3.94 ± 0.97	**
Lean meat percentage (%)	45.64 ± 4.64	63.29 ± 1.97	**
Backfat thickness (cm)	2.49 ± 0.73	2.12 ± 0.61	**
Shear force (N)	23.60 ± 2.16	34.58 ± 6.30	**
Intramuscular fat (%)	5.65 ± 0.78	2.43 ± 0.66	**
Longissimus dorsi muscle area (cm2)	15.89 ± 1.85	58.52 ± 8.77	**

Level of significance: * P < 0.05, **P < 0.01, *NS* not significant.

### Comparison of fatty acid profile and amino acid content in longissimus dorsi muscle of two breeds

We measured the fatty acid composition of the longissimus dorsi muscle in Xiang pigs and Large White pigs (Table 2). We observed 21 fatty acids in two groups that account for 99% of total fatty acids in longissimus dorsi muscle, including eight saturated fatty acids (SFA), five monounsaturated fatty acids (MUFA) and eight polyunsaturated fatty acids (PUFA). The main fatty acids were palmitic acid (C16:0), stearic acid (C18:0), oleic acid (C18:1n9c) and linoleic acid (C18:2n6c), accounting for 90% of total fatty acids in longissimus dorsi muscle, measured palmitic acid (C16:0) and oleic acid (C18:1n9c) in Xiang pigs were significantly higher than that in Large White pigs, while stearic acid (C18:0) and linoleic acid (C18:2n6c) contents of Large White pigs were significantly higher than that in Xiang pigs. Moreover, MUFA content of Xiang pigs was significantly higher than that of Large White pigs (50.73% and 48.83%), and the proportion of PUFA of Xiang pigs was significantly lower than that of Large White pigs (8.49% and 11.26%), the ratio of SFA/UFA of the two breeds were not significant, but it was slightly higher in Xiang pigs than that in Large White pigs.

**Table 2:** Comparison of fatty acid composition of longissimus dorsi muscle in Xiang pigs and Large White pigs (%).

Traits	Xiang pigs (n = 5)	Large White pigs (n = 5)	Level of significance
	Mean ± SD	Mean ± SD	
C10:0	0.10 ± 0.03	0.05 ± 0.00	**
C12:0	0.12 ± 0.03	0.06 ± 0.01	**
C14:0	2.28 ± 0.46	1.05 ± 0.10	**
C16:0	24.72 ± 1.15	23.30 ± 0.89	*
C16:1	0.76 ± 0.18	2.78 ± 0.21	****
C17:0	0.33 ± 0.08	0.12 ± 0.01	**
C18:0	13.03 ± 1.23	15.09 ± 1.28	*
C18:1n9t	1.08 ± 0.20	–	****
C18:1n9c	48.11 ± 0.83	44.96 ± 1.30	**
C18:2n6t	0.02 ± 0.01	–	**
C18:2n6c	7.62 ± 0.88	9.61 ± 0.94	*
C18:3n6	0.01 ± 0.00	–	****
C18:3n3	0.19 ± 0.06	0.20 ± 0.04	ns
C20:0	0.24 ± 0.05	0.17 ± 0.02	*
C20:1	0.40 ± 0.09	0.66 ± 0.09	**
C20:2	0.29 ± 0.07	0.27 ± 0.04	ns
C20:3n6	0.05 ± 0.01	0.10 ± 0.02	**
C20:3n3	0.06 ± 0.01	0.04 ± 0.01	ns
C20:4n6	0.25 ± 0.06	1.06 ± 0.29	***
C22:0	0.01 ± 0.01	0.02 ± 0.00	ns
C22:1n9	0.13 ± 0.08	0.43 ± 0.15	**
SFA	40.84 ± 0.80	39.84 ± 1.92	ns
UFA	58.96 ± 0.79	60.10 ± 1.91	ns
MUFA	50.73 ± 0.78	48.83 ± 1.41	*
PUFA	8.49 ± 1.03	11.26 ± 1.22	**
SFA/UFA	0.69 ± 0.02	0.66 ± 0.05	ns

Level of significance: * P < 0.05, ** P < 0.01, *** P < 0.001, **** P < 0.0001, NS: Not significant.

SFA: Saturated fatty acids, including Capric acid (C10:0), Lauric acid (C12:0), Myristic acid (C14:0), Palmitic acid (C16:0), Margaric acid (C17:0), Stearic acid (C18:0), Arachidic acid (C20:0), Behenic (C22:0).

UFA: Unsaturated fatty acid (MUFA + PUFA).

MUFA: Monounsaturated fatty acids, including Palmitoleic acid (C16:1), Elaidic acid (C18:1n9t), Oleic acid (C18:1n9c), Gadoleic acid (C20:1), Erucic acid (C22:1n9).

PUFA: Polyunsaturated fatty acids, including Linoleilaidic acid (C18:2n6t), Linoleic acid (C18:2n6c), γ-Linolenic acid (C18:3n6), α-Linolenic acid (C18:3n3), cis-11,14-Eicosadienoic acid (C20:2), cis-8,11,14-Homolonolenic acid (C20:3n6), cis-11,14,17-Eicosadienoic acid(C20:3n3), Arachidonic acid (C20:4n6).

We next investigated amino acid content in the longissimus dorsi muscle of Xiang pigs. Among these, glutamate (13.78 ± 0.35), aspartic (7.85 ± 0.15), leucine (7.03 ± 0.15), and lysine (7.69 ± 0.25) had relatively high contents. Compared with the amino acid content of Large White pigs, we found that the content of total amino acids (TAA), delicious amino acids (DAA), and essential amino acids (EAA) were higher in Xiang pigs than in Large White pigs (Table 3).

**Table 3:** Comparison of amino acid content in longissimus dorsi muscle of Xiang pigs and Large White pigs g (100 g)^−1^.

Items	Xiang pigs (n = 5)	Large White pigs (n = 5)	Level of significance
	Mean ± SD	Mean ± SD	
Asp	7.85 ± 0.15	1.56 ± 0.12	****
Thr	3.73 ± 0.11	0.83 ± 0.04	****
Ser	3.18 ± 0.06	0.74 ± 0.05	****
Glu	13.78 ± 0.35	2.88 ± 0.18	****
Gly	3.59 ± 0.18	0.83 ± 0.03	****
Ala	4.84 ± 0.15	1.10 ± 0.06	****
Cys	0.22 ± 0.11	–	****
Val	4.23 ± 0.07	1.00 ± 0.05	****
Met	1.26 ± 0.12	0.42 ± 0.02	****
Ile	4.13 ± 0.21	0.78 ± 0.04	****
Leu	7.03 ± 0.15	1.64 ± 0.10	****
Tyr	2.58 ± 0.08	0.69 ± 0.05	****
Phe	3.82 ± 0.09	0.77 ± 0.04	****
His	3.27 ± 0.12	0.86 ± 0.09	****
Lys	7.69 ± 0.25	1.77 ± 0.11	****
Arg	5.36 ± 0.21	1.30 ± 0.09	****
Pro	–	0.83 ± 0.03	****
TAA	76.56 ± 1.46	17.98 ± 1.09	****
DAA	38.60 ± 0.66	8.41 ± 0.52	****
EAA	31.89 ± 0.88	7.20 ± 0.40	****
DAA/TAA (%)	50.42 ± 0.53	46.77 ± 0.15	****
EAA/TAA (%)	41.65 ± 0.42	40.05 ± 0.21	***

Level of significance: * P < 0.05, ** P < 0.01, *** P < 0.001, **** P < 0.0001, NS: Not significant.

TAA: total amino acid.

EAA: Essential amino acids, including Thr, Val, Met, Ile, Leu, Phe, Lys.

DAA: Delicious amino acids, including Asp, Ser, Glu, Gly, Ala, Arg.

### Summary statistics for RNA-seq data

**Figure 2:**
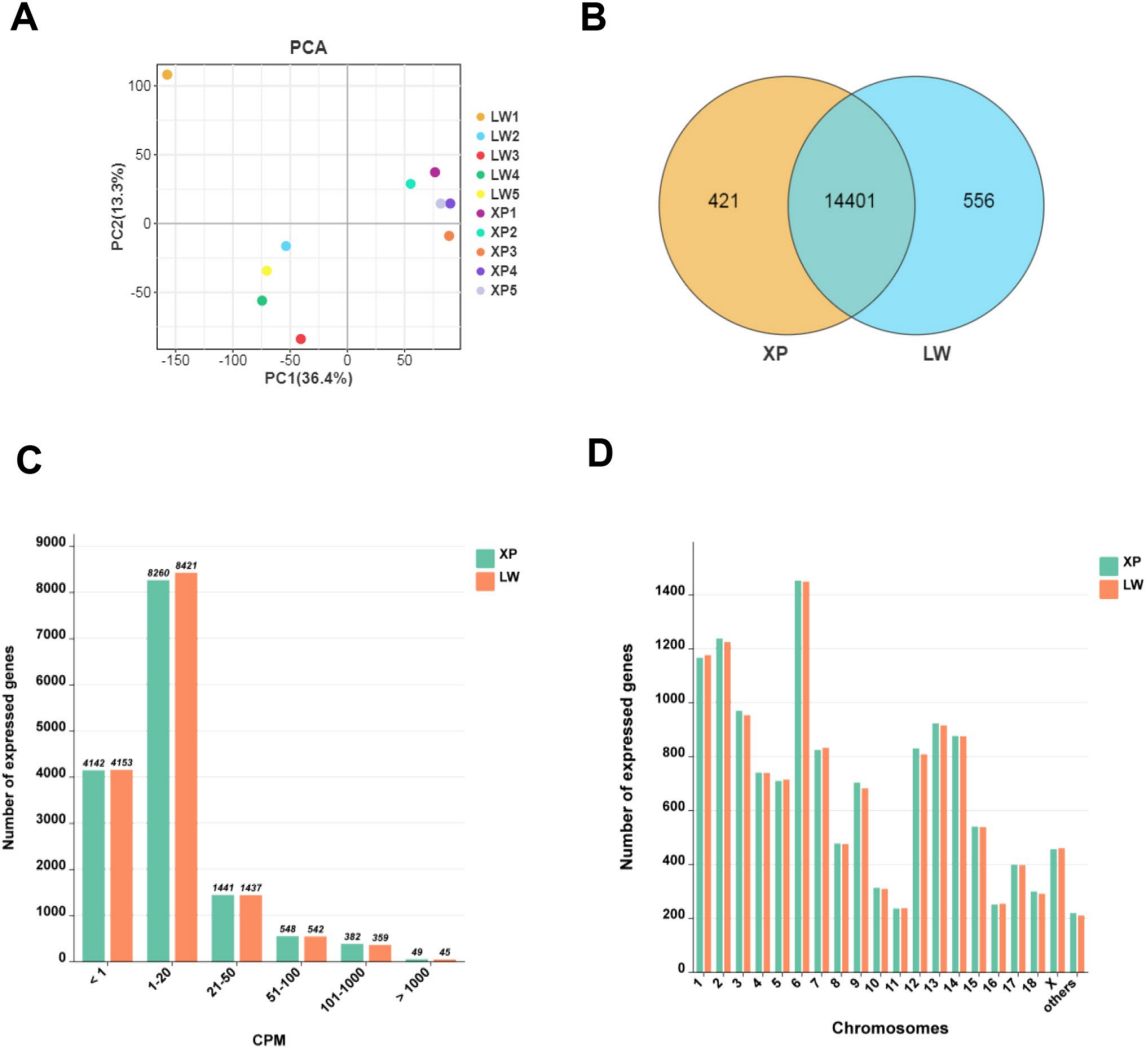

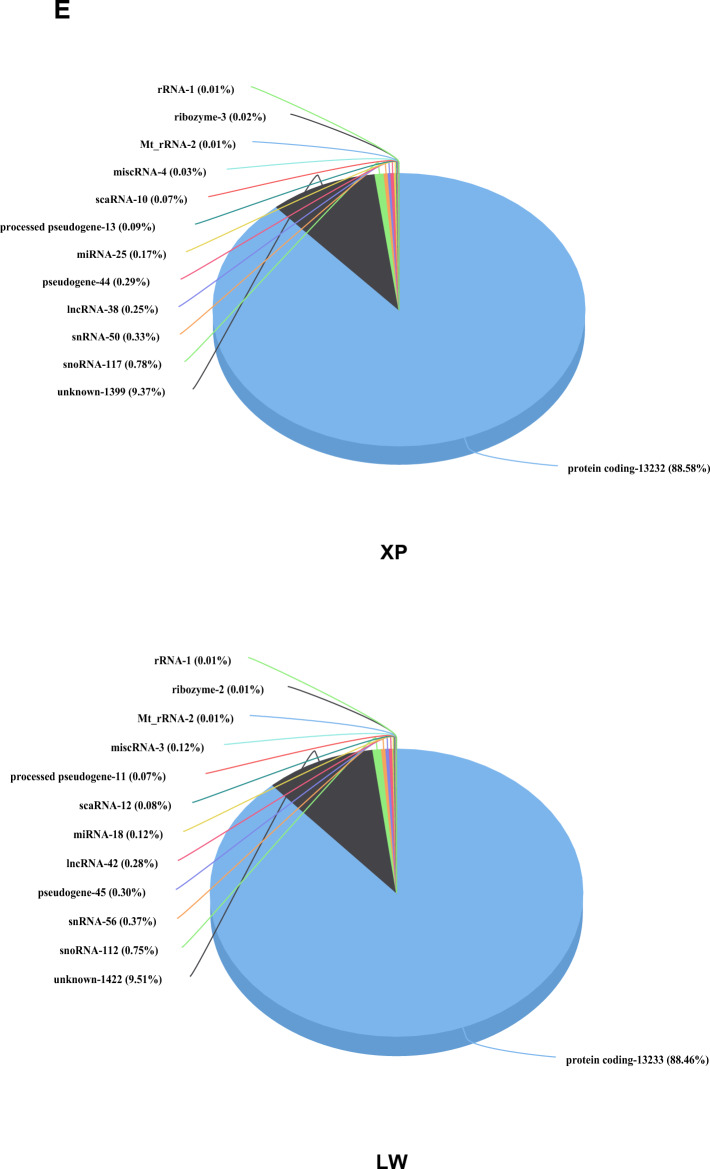
Gene expression analysis of the longissimus dorsi muscle between Xiang pigs and Large White pigs. (**A**) PCA cluster of the gene expression of ten libraries. (**B**) Venn diagram of expression genes in both groups. Yellow color showed genes only expressed in Xiang pig groups, blue color represented genes only expressed in Large White pig groups, and the intersection is the common genes in both groups. (**C**) Gene expression levels in Xiang pig and Large White pig groups. (**D**) Distribution of expressed genes in chromosomes. (**E**) Categories of RNAs in longissimus dorsi muscle of Xiang pigs and Large White pigs.

A total of 1714 million paired-end raw reads were obtained from ten cDNA libraries with 100 base pairs (bp) in length, which yielded 155 million to 187 million raw reads per sample. After filtering adapter sequences and low-quality reads, an average of 169.44 million cleaned reads were produced from the 10 samples. A summary of the sequencing results was shown in Additional Table S4. After alignment, an average of 94.90–98.42% the cleaned reads were mapped to the *Sus scrofa* genome (Sscrofa 11.1), in which 72.42% ~ 81.61% of them were unique matches. PCA plot (Fig. 2A) showed that Xiang pigs and Large White pigs were clearly distinct.

### Comparative transcriptome analysis of longissimus dorsi muscle between Xiang pig and Large White pig breeds

The sequencing data were normalized with counts per million (CPM), a total of 14,822 and 14,957 expressed transcripts were identified including 556 and 421 uncharacterized proteins in Xiang pig and Large White pig groups (Table S5), respectively. Among them, 14,401 genes were expressed in both groups, 421 genes were only expressed in Xiang pigs group, and 556 genes were specifically expressed in Large White pigs group (Fig. 2B). Most of the genes were medium expressed in the longissimus dorsi muscle (1–100 CPM), about 28% of the genes with a low abundance (CPM < 1) (Fig. 2C). Chromosomal distribution analysis revealed a widespread and relatively uniform distribution of expressed genes across all chromosomes, with Chr 1, Chr 2 and Chr 6 being more widely distributed (Fig. 2D).

We investigated the top 30 highly expressed genes in two groups (Table 4), the expression level in Xiang pigs group ranged from 1692.63 to 26,688.19 CPM, which was increased to 1757.51–11,009.65 CPM in Large White pigs group. Of those, including 28 protein-coding genes, and 7 unknown genes. Surprisingly, these highly expressed genes were related to muscle growth and energy metabolism, including actin alpha 1 skeletal muscle (*ACTA1*), nebulin (*NEB*), aldolase fructose-bisphosphate A (*ALDOA*), creatine kinase M-type (*CKM*), glycogen phosphorylase muscle associated (*PYGM*), Cytochrome C oxidase subunit 1 (*COX1*), and ATPase sarcoplasmic/endoplasmic reticulum Ca^2+^ transporting 1 (*ATP2A1*). The enrichment analysis indicated that the top 30 highly expressed genes were significantly enriched (P < 0.05) in pathways related to metabolism and growth, such as glycolysis/gluconeogenesis, biosynthesis of amino acids, carbon metabolism, glucagon signaling pathway, *HIF-1* signaling pathway, pyruvate metabolism, metabolic pathways, and starch and sucrose metabolism (Table S6).

**Table 4:** The top 30 highly expressed level genes in longissimus dorsi muscle of two groups.

Gene ID	Gene name	Gene type	CPM-XP	CPM-LW	Description
ENSSSCG00000010190	ACTA1	Protein_coding	26,688.19349	10,387.22052	Actin alpha 1, skeletal muscle
ENSSSCG00000000694	GAPDH	Protein_coding	13,321.78772	6948.645369	Glyceraldehyde-3-phosphate dehydrogenase
ENSSSCG00000016397	NEB	Protein_coding	12,795.56217	11,009.64976	Nebulin
ENSSSCG00000032556	ALDOA	Protein_coding	8875.221164	5820.337347	Aldolase, fructose-bisphosphate A
ENSSSCG00000036132	CKM	Protein_coding	6581.470674	3902.396333	Creatine kinase, M-type
ENSSSCG00000018003	Unknown	–	6119.776498	6101.460892	–
ENSSSCG00000013022	PYGM	Protein_coding	5983.21115	4052.527227	Glycogen phosphorylase, muscle associated
ENSSSCG00000018075	COX1	Protein_coding	5447.570431	3327.181799	Mitochondrially encoded cytochrome c oxidase I
ENSSSCG00000029441	MYH2	Protein_coding	4423.880987	3689.892706	Myosin, heavy chain 2, skeletal muscle, adult
ENSSSCG00000034677	Unknown	–	3953.806127	3248.149629	–
ENSSSCG00000007805	ATP2A1	Protein_coding	3900.626852	1912.571089	ATPase sarcoplasmic/endoplasmic reticulum Ca2 + transporting 1
ENSSSCG00000019556	Unknown	–	3824.764003	2861.341169	–
ENSSSCG00000026098	CMYA5	Protein_coding	3511.598671	2719.687879	Cardiomyopathy associated
ENSSSCG00000016578	FLNC	Protein_coding	3350.218007	3572.723628	Filamin C
ENSSSCG00000038561	Unknown	–	3308.748682	3244.026808	–
ENSSSCG00000016157	MYL1	Protein_coding	3272.679751	2893.378725	Myosin light chain 1
ENSSSCG00000001930	PKM	Protein_coding	3226.112413	1855.691812	Pyruvate kinase, muscle
ENSSSCG00000004570	TPM1	Protein_coding	3040.082186	3044.597925	Tropomyosin 1
ENSSSCG00000017904	ENO3	Protein_coding	2920.622911	2906.914686	Enolase 3
ENSSSCG00000003218	MYBPC2	Protein_coding	2509.413998	1648.19752	Myosin binding protein C2
ENSSSCG00000037545	Unknown	–	2408.878332	2175.830853	–
ENSSSCG00000012699	FHL1	Protein_coding	2292.269637	1598.086628	Four and a half LIM domains 1
ENSSSCG00000018082	COX3	Protein_coding	2256.369716	1497.590602	Cytochrome c oxidase subunit III
ENSSSCG00000031740	Unknown	–	2137.980162	7535.428967	–
ENSSSCG00000013366	LDHA	Protein_coding	2073.378807	1527.340768	Lactate dehydrogenase A
ENSSSCG00000015917	XIRP2	Protein_coding	2065.485058	2829.131003	Xin actin binding repeat containing 2
ENSSSCG00000005316	TPM2	Protein_coding	1972.892121	2091.84458	Tropomyosin 2
ENSSSCG00000002029	MYH6	Protein_coding	1948.309282	2802.028952	Myosin-6
ENSSSCG00000020785	DES	Protein_coding	1845.302448	1967.990019	Desmin
ENSSSCG00000032395	Unknown	–	1692.627707	102.1836673	–
ENSSSCG00000040929	TPT1	Protein_coding	1464.14554	2557.365322	Tumor protein, translationally-controlled 1
ENSSSCG00000015796	PDLIM3	Protein_coding	1231.64966	1909.184688	PDZ and LIM domain 3
ENSSSCG00000004725	ZNF106	Protein_coding	1301.557693	1905.562842	Zinc finger protein 106
ENSSSCG00000036948	IGFN1	Protein_coding	1602.924074	1858.54288	Immunoglobulin like and fibronectin type III domain containing 1
ENSSSCG00000010304	MYOZ1	Protein_coding	1461.882701	1757.505806	Myozenin 1

According to their biogenesis and annotation, all expressed genes were divided into different categories of RNAs. Our annotation results showed that the same trend of expressed genes presented in the two groups, the largest fractions were the protein coding genes (88.50% and 88.46%), followed by the un-annotated genomic regions (9.37% and 9.50%). We also observed that the proportion of miRNAs in the Xiang pigs group was significantly higher than that in the Large White pigs group (8.14% vs 5.92%), and the proportion of snRNA in Xiang pigs group was slightly lower than that in the Large White pigs group (16.29% vs 18.42%). The other non-coding RNAs (lncRNA, miscRNA, scaRNA, snoRNA) were almost equally distributed in the two groups (Fig. 2E).

**Figure 3:**
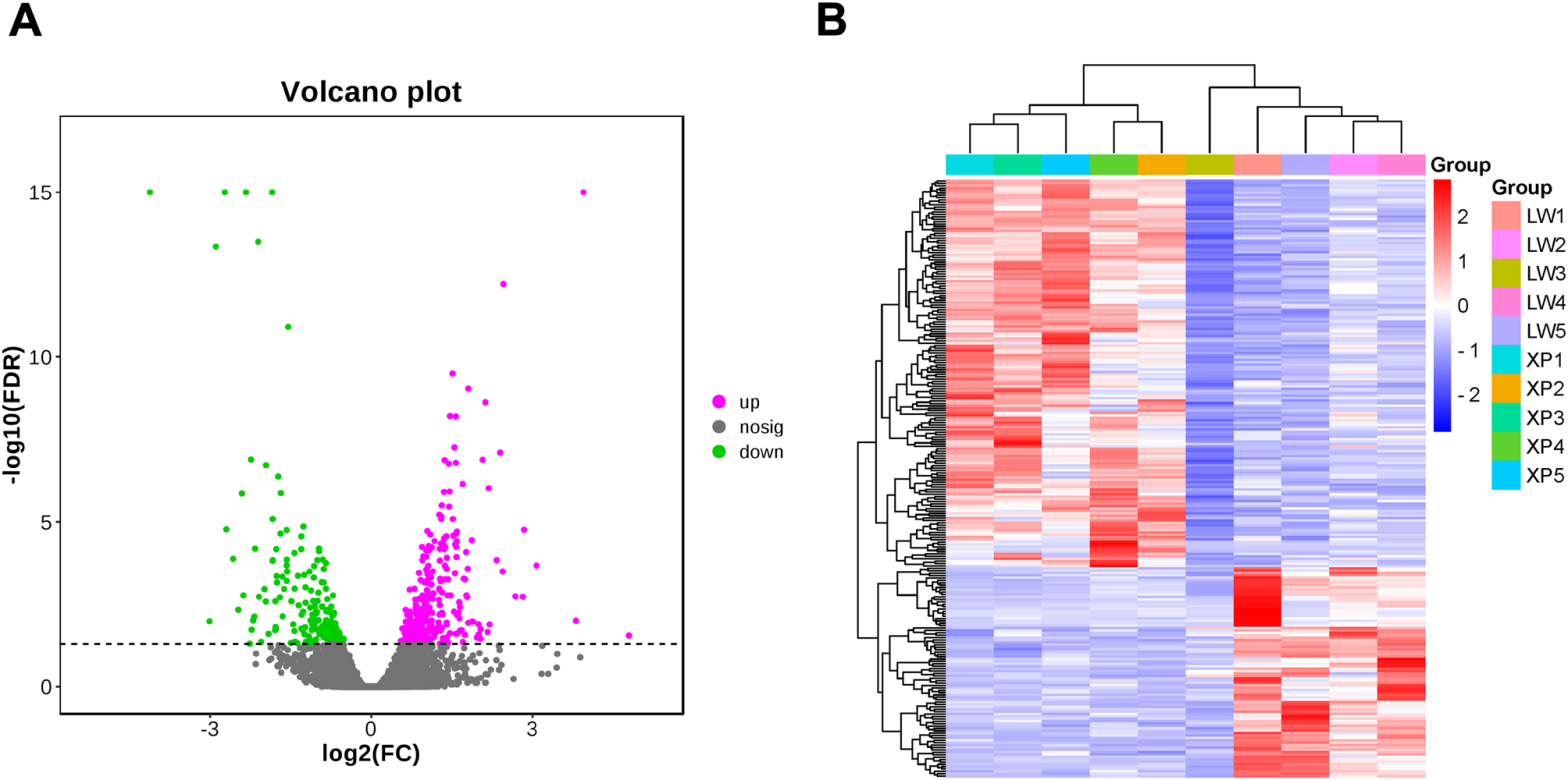
Differential expression analysis. (**A**) Volcano plots of the DEGs. Each point in the figures represented one gene. Pink points represented up-regulated genes, green points denoted down-regulated genes. Grey points were genes without significant difference. (**B**) Heat map of 251 DEGs expression levels between Xiang pigs and Large White pigs.

After removing those genes with CPM < 1.0 in each sample, noncoding RNA, and pseudogene transcripts, the normalizing read data of the protein genes were used for differential profile analyses between Xiang pigs and Large White pigs. A total of 251genes were differently expressed between the two groups, and 163 and 88 genes were up-regulated and down-regulated in the Xiang pig group, respectively. The detailed information was provided in Table S7. The Fig. 3 showed the volcano diagram and heatmap of these DEGs. In addition, among the TOP 30 most abundant DEGs, a lot of genes were involved in meat quality trait, such as *ACTA1, ATP1A2, HSP90AA1, TNNC1, TNNT1, ATP6, SLC2A4, CARNS1,* and *PFKFB3.*

### Functional analysis of DEGs involved in meat quality traits

**Figure 4:**
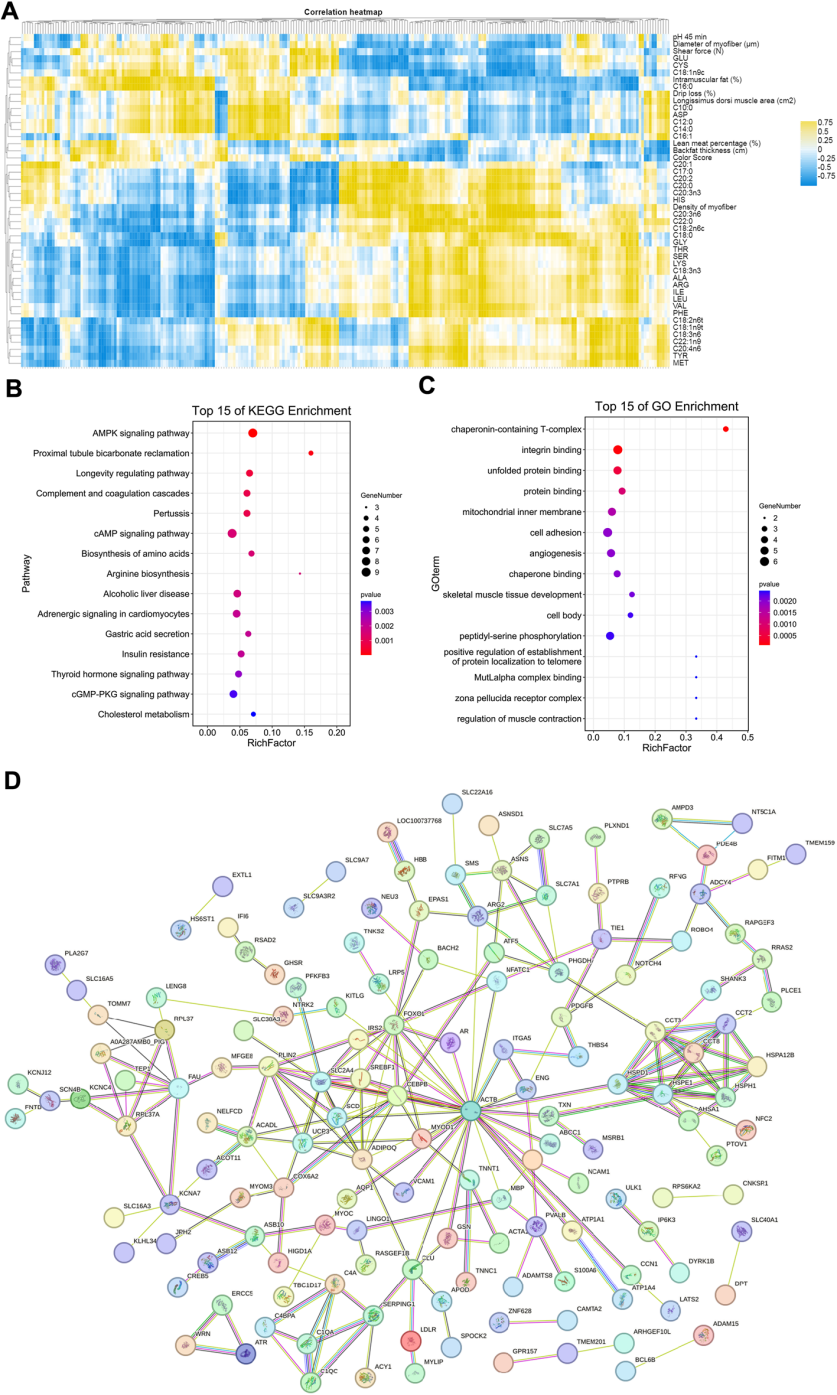

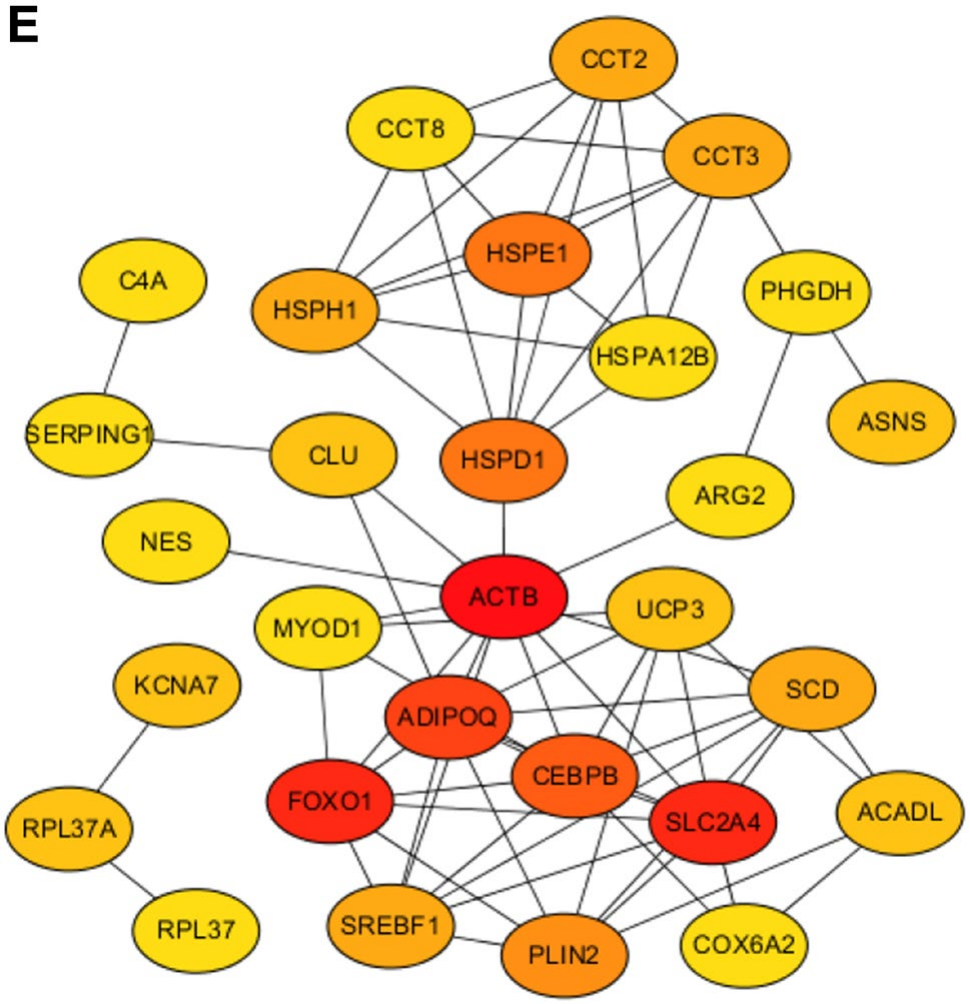
Functional analysis of DEGs of the longissimus dorsi muscle. (**A**) Pearson correlation analysis of 251 differentially expressed genes and meat quality traits. (**B**) The top 15 KEGG enrichment analysis of 227 DEGs. (**C**) The top 15 Go functional analysis of 227 DEGs. (**D**) The PPI network consisted of 148 DEGs. (**E**) The PPI network of the 29 hub genes.

We performed the Pearson correlation analysis between the DEGs and meat quality values in the data of 10 pig samples measured in this study (Fig. 4A). A total of 251 genes were significantly correlated with meat quality indicators, fatty acids, and amino acids (P < 0.05). The detailed information of these significantly correlated DEGs was presented in Table S8. Furthermore, we compared the DEGs with the QTLs collected in the pig QTL database (http://www.animalgenome.org/cgi-bin/QTLdb/SS/index, Release 50, Apr 25, 2023). In total, 227 DEGs were mapped to the 214 meat quality QTLs (Table S9). High proportion of DEGs was significantly enriched in QTLs affecting loin muscle area (175 DEGs), muscle pH (157 DEGs), backfat at last rib (144 DEGs), average backfat thickness (130 DEGs), drip loss (106 DEGs), and meat color score (51 DEGs).

Combining the above two filtering criteria, we screened out 227 DEGs that their expressions were significantly correlated with meat quality values in Xiang pigs, and also located in the QTLs of meat quality trait. To have an insight of function for these DEGs, we performed KEGG and GO enrichment analysis (Fig. 4B,C) (Table S10). KEGG enrichment analysis revealed that these DEGs were mainly enriched in the pathways of cell proliferation and energy metabolism regulation (P < 0.01), such as aldosterone synthesis and secretion, thyroid hormone signaling pathway, aldosterone-regulated sodium reabsorption, gastric acid secretion, AMPK, cAMP, cGMP-PKG, biosynthesis and metabolism of amino acid and Insulin resistance pathways. The GO analysis determined 42 significantly enriched GO terms (P < 0.01). Most of them were involved in developmental process, cell differentiation, metabolic process, regulation of biological process, myofibril, contractile fiber, sarcomere, supramolecular fiber, supramolecular polymer, supramolecular complex, organelle, and intracellular organelle. We further analyzed the protein–protein interaction (PPI) between the 227 DEGs using the Search Tool for the Retrieval of Interacting Genes (STRING). In total, 148 of 227 DEGs could be constructed PPI network (Fig. 4D). Of them, 29 genes were identified to be hub genes based on the coexpressed genes with ≥ 10.0 degrees (Fig. 4E) (Table 5). The genes with the highest network weights included *MYOD1, ACTB, ASNS, FOXO1, ARG2, SLC2A4, PLIN2,* and *SCD*, which may play important roles in meat quality.

**Table 5:** The detailed information of the hub genes.

Gene information		logFC	Gene description	CPM-XP	CPM-LW	Node_degree
ID	Gene name					
ENSSSCG00000007585	ACTB	1.067809913	Actin beta	183.0535706	85.32924318	52
ENSSSCG00000009370	FOXO1	1.079991322	Forkhead box O1	29.65339591	14.0482641	24
ENSSSCG00000023915	SLC2A4	1.157657422	Solute carrier family 2 member 4	120.3156739	51.30256281	24
ENSSSCG00000039103	ADIPOQ	− 2.230867066	Adiponectin, C1Q and collagen domain containing	7.940576274	40.77274086	22
ENSSSCG00000034207	CEBPB	1.448520239	CCAAT enhancer binding protein beta	85.040787	30.91433198	20
ENSSSCG00000016078	HSPE1	− 1.687154846	Heat shock protein family E (Hsp10) member 1	9.850477548	32.55719681	18
ENSSSCG00000016077	HSPD1	− 1.051522015	heat shock protein family D (Hsp60) member 1	21.949064	46.97844214	18
ENSSSCG00000035863	PLIN2	− 1.266860216	Perilipin 2	17.31239955	41.6470245	16
ENSSSCG00000006487	CCT3	− 1.050775945	Chaperonin containing TCP1 subunit 3	32.31517486	71.09025048	14
ENSSSCG00000000496	CCT2	− 1.124582976	Chaperonin containing TCP1 subunit 2	23.47270217	53.18409378	14
ENSSSCG00000009334	HSPH1	− 1.302812287	Heat shock protein family H (Hsp110) member 1	25.885635	70.24457978	14
ENSSSCG00000033626	SREBF1	1.538998761	Sterol regulatory element binding transcription factor 1	30.17292329	9.845588543	14
ENSSSCG00000010554	SCD	1.571969711	Stearoyl-CoA desaturase	32.58577776	11.448515	14
ENSSSCG00000015340	ASNS	1.880263609	Asparagine synthetase (glutamine-hydrolyzing)	23.06212025	6.128967355	12
ENSSSCG00000009668	CLU	− 1.273062166	Clusterin	11.62206076	29.01003306	12
ENSSSCG00000003159	KCNA7	1.403184952	Potassium voltage-gated channel subfamily A member 7	17.13146379	6.39130437	12
ENSSSCG00000014834	UCP3	1.769200841	uncoupling protein 3	93.5853444	27.30302368	12
ENSSSCG00000016156	ACADL	− 1.15172263	Acyl-CoA dehydrogenase long chain	14.99175262	32.85702425	12
ENSSSCG00000036716	RPL37A	− 1.062799068	Ribosomal protein L37a	12.99983522	27.18312858	12
ENSSSCG00000026761	CCT8	− 1.056343738	Chaperonin containing TCP1 subunit 8	19.37960105	42.08100745	10
ENSSSCG00000007147	HSPA12B	1.01283593	Heat shock protein family A (Hsp70) member 12B	12.30241643	6.026123472	10
ENSSSCG00000001427	C4A	− 2.181772223	Complement C4A (Rodgers blood group)	4.897857848	23.63926045	10
ENSSSCG00000006717	PHGDH	3.81011071	Phosphoglycerate dehydrogenase	4.367714421	0.192734135	10
ENSSSCG00000013181	SERPING1	− 1.097663124	Serpin family G member 1	20.30967255	44.00003521	10
ENSSSCG00000006474	NES	− 2.574875041	Nestin	14.56541875	98.87398386	10
ENSSSCG00000013375	MYOD1	1.294236262	Myogenic differentiation 1	12.35289749	4.930615012	10
ENSSSCG00000002294	ARG2	3.07559455	Arginase 2	16.29654104	1.810641781	10
ENSSSCG00000036789	COX6A2	1.145570188	Cytochrome c oxidase subunit 6A2	51.59934718	22.71131411	10
ENSSSCG00000033326	RPL37	− 0.96548223	Ribosomal protein L37	6.89885077	13.57159637	10

**Figure 5:**
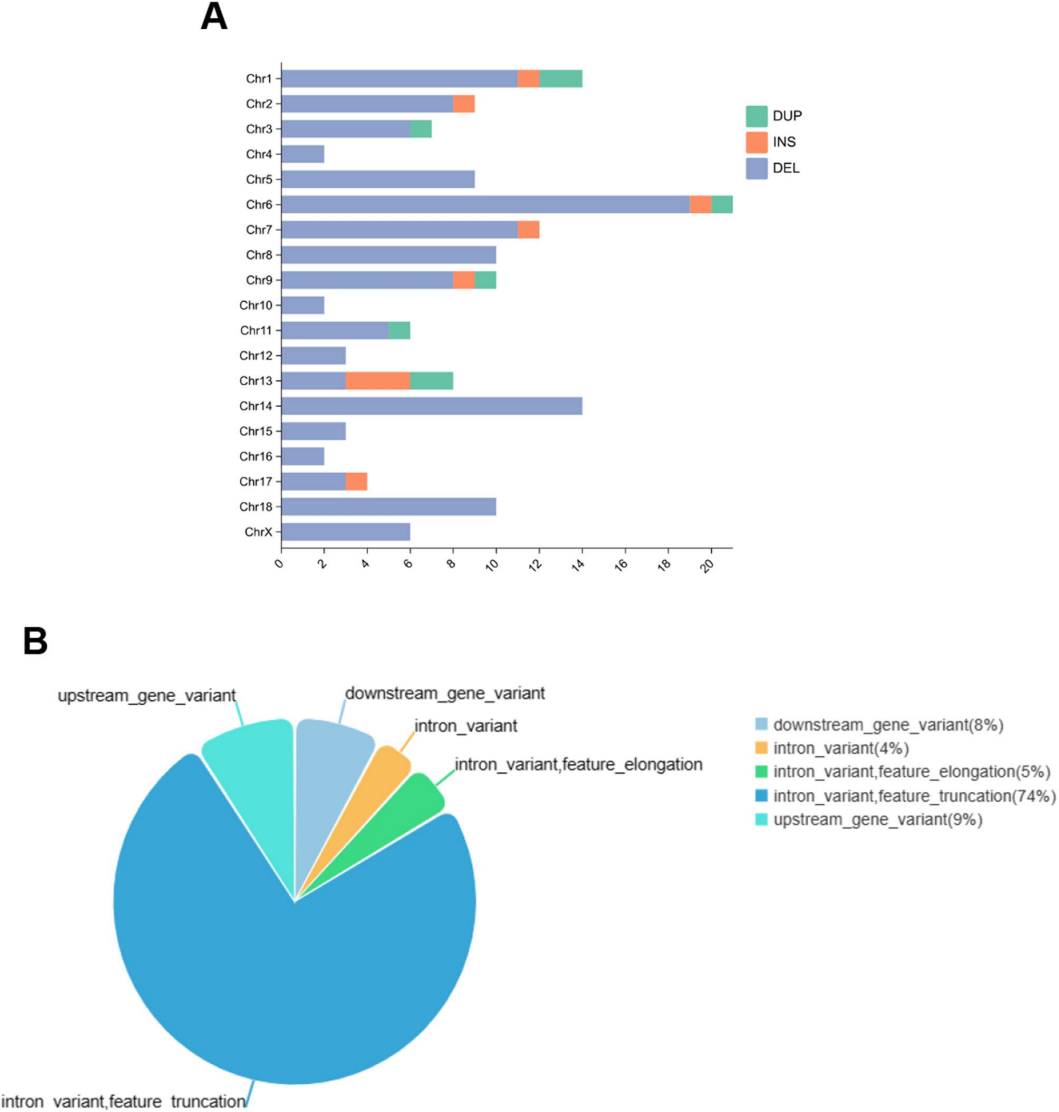
The distribution of SVs. (**A**) Distribution of the three variant types on chromosomes. (**B**) Distribution of variant types on gene regions.

We further collected genetic variation between Xiang pigs and Western commercial pigs previously reported by our group^29^, and we found 75 DEGs covering 152 loci of structural variation (SV), which deletion accounted for the major type of variation and most of the variations were located in introns (Table S11, Fig. 5A,B). We also observed structural variation in some hub genes, such as a 251-bp deletion on Chromosome 15 (15:112,931,020–112,931,271) in the downstream of the *ACADL* gene exhibited SV distributed among 9 Xiang pigs and 1 Large White pig, as well as the deletion of 103 bp on Chromosome 14 (15:111,480,601–111,480,704) in the downstream of the *SCD* gene presented in 11 European pig breeds. These variants could have an important effect on the level of gene expression.

### Tests and verification

**Figure 6:**
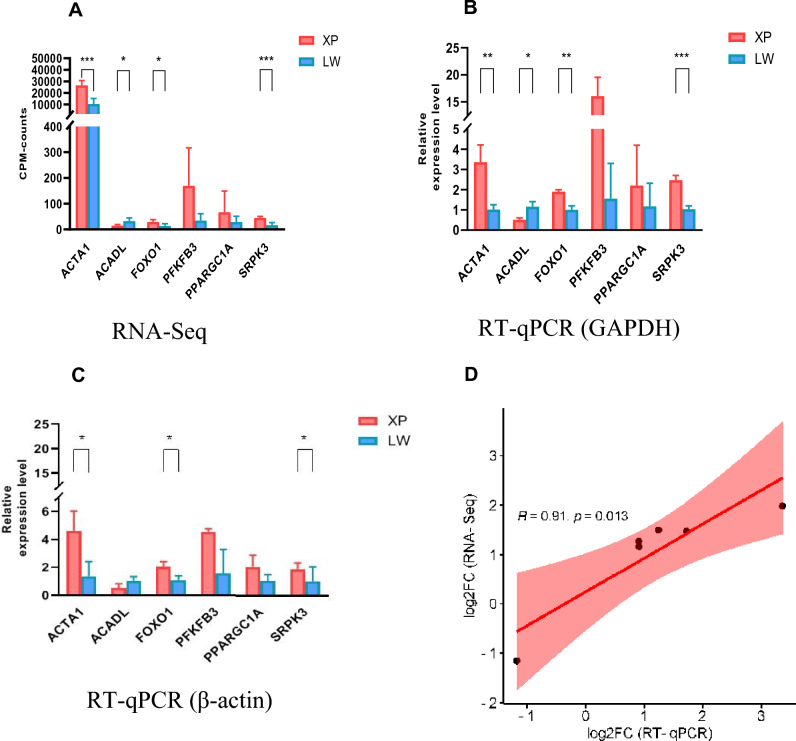
Validation of RNA-seq results by RT-qPCR. (**A**) RNA-seq results of the six selected DEG in of the six selected DEG in Xiang pigs and Large white pigs group. (**B**) Relative expression level verified by *GAPDH* of the 6 selected DEG in Xiang pigs and Large white pigs group. (**C**) Relative expression level verified by β-actin of the 6 selected DEG in Xiang pigs and Large white pigs group. (**D**) The trend was similar in log2FC (XP/LW) from RNA-seq and the ratio of expression levels in groups by RT-qPCR method with the linear correlation coefficient of R^2^ = 0.8281, P = 0.0013 based on two-tail T-test.

To validate the RNA-seq data, six genes (*ACTA1, ACADL, FOXO1, PFKFB3, PPARGC1A, SRPK3*) were randomly selected and confirmed by RT-qPCR. The results showed a similar trend between those measured by RT-qPCR and RNA-seq analysis (Fig. 6A–D), indicating that the analysis based on RNA-seq data was precise and effective.

## Discussion

In pig industry, meat quality is one of the most important economic traits in the world, which is influenced by multifactor including heredity and environment^30^. Xiang pig is one of the native pig breeds in China, with small body and excellent meat quality characteristics. Previous studies have indicated that the meat quality of Chinese local pig breeds is better than Western commercial pigs, such as Large White, which is a commercial breed with a fast growth rate and poor meat quality^16,22,31^. In our study, we found that obvious differences in carcass and meat quality traits between Xiang pigs and Large White pigs, such as pH, color, drip loss and shear force. In addition, Xiang pigs group had a higher backfat thickness, and IMF content than Large White pigs group, while the lean meat percentage, and loin muscle area were lower in Xiang pigs group than in Large White pigs group. These results indicated that fat deposition is greater than fat decomposition in Xiang pig, compared to Large White pigs.

In order to identify candidate genes that might affect the meat quality of Xiang pigs, we selected the longissimus dorsi muscle samples from Xiang pig and Large White pig in the same environment, and carried out comparative analysis of the expression profile. A total of 251 DEGs were identified between Xiang pigs and Large White pigs groups. To further obtain DEGs affecting meat quality, correlation analysis and QTLs analysis were performed. Consequently, we screened out 227 critical DEGs affecting meat quality, and whose expressions were significantly correlated with meat quality values in Xiang pigs, and these DEGs were mapped to 214 meat quality QTLs, such as backfat thickness, IMF content, lean meat percentage, drip loss, shear force, muscle fibers, fatty acid content, and tenderness score. KEGG enrichment analysis revealed that these 227 DEGs were significantly enriched in 20 signaling pathways (P < 0.01), most of which were involved in energy metabolism and regulation cell proliferation, such as cGMP-PKG signaling pathway, insulin resistance, regulation of lipolysis in adipocytes, AMPK signaling pathway, and biosynthesis and metabolism of amino acid. Most of GO terms were involved in developmental process, cell differentiation, metabolic process, regulation of biological process, myofibril, contractile fiber, organelle, and intracellular organelle, which were similar to the studies for local pig breeds^2,4,32^. It indicated that these DEGs might mediate the meat quality traits difference between Xiang pigs and Large White pigs. Moreover, of the 148 DEGs would be constructed PPI network, consisting of 29 core genes (Table 5). In addition, we further collected previous studies that found some important SVs affecting the expression of some key genes in Xiang pigs, such as *SCD, GPCPD1, PLA2G7, ACADL,* and *PLIN2.* These crucial DEGs would provide key information for understanding the molecular mechanisms of meat quality.

The deposition of fat in animals represents the balance between fat synthesis and catabolism. Once the original balance is disrupted, fat deposition increases or body fat decreases, thereby affecting the meat quality of animals^33^. Traditionally, Xiang pigs are raised on small family farms, with a small number of sows fed on household food residues and horticultural by-products or green fodder, which leads to excessive calorie intake and is beneficial for fat generation and accumulation rather than catabolic pathways. Our transcriptome analysis indicated that the genes, such as *IRS2, FOXO1,* and *GLUT4*, involved in the insulin signaling response were also more highly expressed in the longissimus dorsi muscle of Xiang pigs than in Large White pigs, which is consistent with previous studies that up regulated genes involved in insulin signaling pathway and insulin resistance in local pig breeds compared to western commercial pigs^14,34^. Interestingly, we observed that some genes harboring SV were enriched in the insulin signaling pathway^29^, including *RAPGEF1* (Chr1:271,538,137–271,538,226, 89 bp-DEL), *PYGL* (Chr1:180,715,345–180,715,345, 71 bp-INS), and *MTOR* (Chr6:71,407,644–71,407,932, 288 bp-DEL), and these variants (INS and DEL) appeared more frequently in Xiang pigs than in Large White pigs, suggesting that variation in these genes may leads to high expression of related genes in the insulin signaling related pathway in Xiang pigs. In addition, the *PFKFB3* gene regulates the synthesis and degradation of fructose-2, 6-bisphosphate and fructose-6-phosphate during glucose metabolism. Previous studies suggest that *PFKFB3* may be involved in fat deposition and regulation mediated by the glycogen pathway^35^. In our study, we found that *PFKFB3* was highly expressed in the Xiang pigs group and significantly enriched in the AMPK signaling pathway. A study on several Chinese breeds as compared to Yorkshire breeds revealed upregulated expression of genes associated with lipid metabolic process and fatty acid biosynthetic process, and the expression of genes involved in fat oxidation was lower^36,37^. In addition, similar studies have reported higher expression of genes related to fatty acid, lipid and phospholipid synthesis in local Basque pigs compared to Large White pigs, whereas the Wujin breed showed lower expression of lipase E (*LIPE)* and adipose tissue triglyceride lipase compared to Landrace pigs, suggesting that the Wujin breed has lower lipolysis, higher lipogenesis and better fatty acid transport^38,39^. It can be concluded that the expression of lipogenic genes and fatty acid transport genes was higher, or to a higher rate of adipogenesis, while genes related to lipid mobilization and expenditure were down-regulated in the in local breeds compared to modern breeds. Meanwhile, the expression of genes involved in lipogenesis, such as *SCD, GPCPD1, CEBPB,* were also up regulated and the genes related to the lipolysis, such as *ACADL, PLIN2*, *LIPA* were down regulated in the muscle of Xiang pigs compared to Large White pigs. Thus, our results suggested that Xiang pigs could secrete more insulin for controlling circulating glucose levels by inducing glucose uptake and storage as glycogen, while triggering upregulation of lipogenic and adipogenic processes. Interestingly, our previous study found that these genes (*GPCPD1, ACADL, PLIN2,* and *LIPA*) had a high frequency of DEL in the Xiang pig group (Table S11)^29^. Notably, we found an 103 bp deletion was found in downstream of *SCD* gene in European pig breeds, which resulted in the loss of the ssc-miR-3331-3p binding site based on miRbase. Ultimately, the variation in these genes may have led to differential expression in Xiang pigs and Large White pigs.

The quality and yield of meat are determined by the development and growth of muscle. Previous researchers have found that meat quality was related to muscle fiber characteristics and IMF content^22^. Muscle fiber traits are closely related to meat color, water holding capacity, pH, tenderness, and IMF content. We found that the pH 45 min, color score, and muscle fiber density in Xiang pigs were higher than that in Large White pigs, while the lean meat percentage, longissimus dorsi muscle area, and muscle fiber diameter in Xiang pigs were lower than that in Large White pigs, which was consistent with the lower shear force and more tender in Xiang pigs. This is more similar to other local pig breeds in China^40–42^. Some genes were involved in muscle development, such as *MYOD1, MYLK4, ASNS, CARNS1,* and *PVALB,* these genes were significantly up-regulated in the longissimus dorsi muscle of Xiang pigs, and *MYLIP, TNNC1, MYOM3, MYOC,* and *TNNT1,* were also significantly down-regulated in longissimus muscle of Xiang pigs. Therefore, the roles of these genes in the regulation of muscle development were the rationale for the reduced leanness of Xiang pigs compared to Large White pigs. In addition, we found 210 DEGs mapped to 17 QTLs associated with muscle development, including loin muscle area (175 DEGs), muscle pH (157 DEGs), muscle conductivity (85 DEGs), and total muscle fiber number (7 DEGs). The *MYOC* is an important gene that regulates the size of skeletal muscle fibers and causes an increase in muscle fiber diameter when expression is activated, and causes conditions including atrophy of muscle fibers, fragility of muscle membranes and impaired muscle regeneration to occur if loss of *MYOC*^43,44^. Serine/arginine protein kinase (*SRPK*), a family of cell cycle regulated protein kinases, *SRPK3* mRNA expression level in embryonic is important for the determination of muscle fiber development and muscle fiber number, its expression level decreases slowly after birth^45^. We found that the *MYOC* gene was significantly down-regulated and *SRPK3* was significantly up-regulated in Xiang pigs. This may be a factor contributing to the difference in muscle fiber density and diameter between Xiang pigs and Large White pigs. Muscle development is regulated by various signaling pathways. Our KEGG pathway analysis revealed some classical pathways involved in the regulation of muscle development, such as adrenergic signaling in cardiomyocytes, thyroid hormone signaling pathway, cardiac muscle contraction, and AMPK signaling pathway. Interestingly, AMPK plays a key regulatory role in muscle energy metabolism and is closely related to muscle fiber type conversion. In addition, previous studies have shown that the *GLUT*4 is inhibited in skeletal muscle cells, leading to metabolic disorders and skeletal muscle atrophy^46^. In our study, we found that *GLUT*4 was significantly up-regulated in Xiang pigs and was significantly enriched in the AMPK signaling pathway, which provided the basis for the difference in muscle fibers between Xiang pigs and Large White pigs.

The composition of fatty acids plays an important role in the development of the flavor of pork. Many fatty acids react to form volatile aromatic substances during the cooking process. In particular, MUFA has a positive impact on the development of meat flavor, while PUFA inhibits the degradation pathways of Maillard and thiamin, thus affecting the overall flavor formation^47–49^. Furthermore, the higher PUFA content leads to higher reduction in muscle water holding capacity and reduced muscle juiciness, and PUFA is more easily oxidized than SFA, it affects the associated aroma and flavor^50,51^. Most of the fatty acids showed a high correlation with androgens. MUFA content was negatively correlated with androstenone and skatole content, while PUFA was positively correlated with androstenone^52^. In our research, we found that Xiang pigs had higher MUFA content, while PUFA content was significantly lower than that of Large White pigs. Similarly, previous studies have shown that MUFAs are predominant in Chinese native pigs^53^. Differential expression analysis showed that *SCD* was up regulated and *ACADL* was down regulated in the muscle of Xiang pigs compared to Large White pigs, and enriched in fatty acid metabolism, unsaturated fatty acid biosynthesis, fatty acid degradation pathways. The expression of these genes could be related with the high MUFA and low PUFA content in Xiang pigs. Furthermore, previous studies have reported that palmitic acid (C16:0) and stearic acid (C18:0) had a particularly important effect on the firmness of adipose tissue, and the ratio of SFA/UFA was the best ratio for predicting fat hardness, and have found that fishy flavor is positively correlated with alpha-linolenic acid and docosahexaenoic acid in pork^54,55^. In addition, we found that the ratio of SFA/UFA in longissimus dorsi muscle of Xiang pigs was higher and the alpha-linolenic acid content was lower than Large White pigs in this study, this suggests that Xiang pigs have firmer fat and less fishy flavor compared to Large White pigs.

Amino acids affect freshness and flavor and are a good indicator of protein content in muscle^4^. In our study, we found that TAA, EAA, and DAA were more abundant in longissimus dorsi muscle of Xiang pigs, which was similar to the result of previous studies^56^. Functional enrichment analysis revealed that some DEGs were enriched in biosynthesis of amino acids pathway, such as *ASNS*, *ARG2*, *ACY1*, *PHGDH*, and *CARNS1*, were up regulated and the *SMS* gene was down regulated, it leads to the accumulation of fresh-flavored amino acids (including alanine, aspartate, glutamate, glycine, arginine, and lysine) in the longissimus dorsi muscle of Xiang pigs compared to Large White pigs. In addition, *SLC7A1* plays an important role in the transport of cationic amino acids in porcine muscle tissues, and previous studies have shown that the high expression of *SLC7A1* in JXB pigs may be associated with their high levels of cationic amino acids (lysine, arginine, and histidine)^57^. In our study, we found that the *SLC7A1* gene was likewise highly expressed in Xiang pigs. Thus, these DEGs further explained the difference in amino acid content in the longissimus dorsi muscle between Xiang pigs and Large White pigs.

Muscle is composed of muscle fiber and water, and water content directly affects the tenderness of muscle. In this study, we found that drip loss and shear force of Xiang pigs were lower than Large White pigs, which indicated better water retention and more tender meat for Xiang pigs. In addition, we found a number of DEGs mapped to QTLs related to drip loss (106 DEGs) and water holding capacity (24 DEGs), such as *ATP1A1, MYOD1, SLC2A4,* and *HSPE1.* Previous studies have shown that Na^+^/K^+^-ATPase and solute carrier family genes (*SLC*) are essential for maintaining transmembrane Na^+^ and K^+^ gradients and water homeostasis, and the genes of solute carrier family (SLC) have been reported to be promising candidate genes influencing drip loss trait^58–60^. In our study, *ATP1A2* and *SLC16A3* were significantly up-regulated, and *ATP1A1* was down-regulated in Xiang pigs. Moreover, these genes (*ATP1A2*, *ATP2B3*, *ATP1A1*, and *SLC38A3*) were enriched in pathways highly associated with muscle water retention, including proximal tubule bicarbonate reclamation, thyroid hormone signaling pathway, aldosterone synthesis and secretion, and aldosterone-regulated sodium reabsorption. In addition, previous studies have identified *ITGA5* genes are significantly associated with drip loss and water holding capacity, and *ITGA5* was up-regulated in the low drip loss group^61,62^, which is consistent with our differential expression results. Except potential candidate genes listed above, heat shock proteins (*HSPs*) have been reported as potential biomarkers affecting water holding capacity and tenderness^63,64^. In this study, several *HSP* genes, like *HSPA12B*, *HSPE1*, *HSPH1*, and *HSPD1* were found to differently express between the Xiang pigs and Large White pigs groups. Hence, these genes provide a better understanding of the differences in drip loss and water holding capacity between Xiang pigs and Large White pigs, and further studies should be carried out to unravel their specific mechanism in drip loss and water holding capacity.

The present study concluded with the superiority of Xiang pigs to Large White pigs in meat traits and flavor substances. Through transcriptome analysis, we identified 163 genes that showed up-regulated and 88 genes that appeared to be down-regulated in longissimus muscle of the Xiang pigs. Function enrichment analysis revealed that DEGs were enriched in several meat quality-related signaling pathways, such as aldosterone synthesis and secretion, thyroid hormone signaling pathway, aldosterone-regulated sodium reabsorption, gastric acid secretion, AMPK, cAMP, cGMP-PKG, biosynthesis and metabolism of amino acid and nsulin resistance pathways. Based on Pearson correlation analysis, QTL localization and Protein Interaction Analysis, a total of 29 hub genes were found to be corresponding to the meat quality in Xiang pigs. These genes play important roles in cell proliferation, energy metabolism regulation, and development of muscle and fat. This study explains the reasons for the differences in meat quality traits and flavor substances between the two pig strains. It provides a theoretical basis for in-depth research on Xiang pigs and utilization of Chinese local pigs to obtain high-quality meat pig strains and new breeds.

### Supplementary Information

Supplementary Table 1.

Supplementary Table 2.

Supplementary Table 3.

Supplementary Table 4.

Supplementary Table 5.

Supplementary Table 6.

Supplementary Table 7.

Supplementary Table 8.

Supplementary Table 9.

Supplementary Table 10.

Supplementary Table 11.

Supplementary Figure 1.

## Data Availability

The data supporting the conclusions of this article are included within the article and its addition files.
