# Supplementary figures and images for: Comparative transcriptome analysis of longissimus dorsi muscle reveal potential genes affecting meat trait in Chinese indigenous Xiang pig
Source: Sci Rep. 2024 Apr 11;14:8486. doi: 10.1038/s41598-024-58971-2 (PMC11009340; doi:10.1038/s41598-024-58971-2)

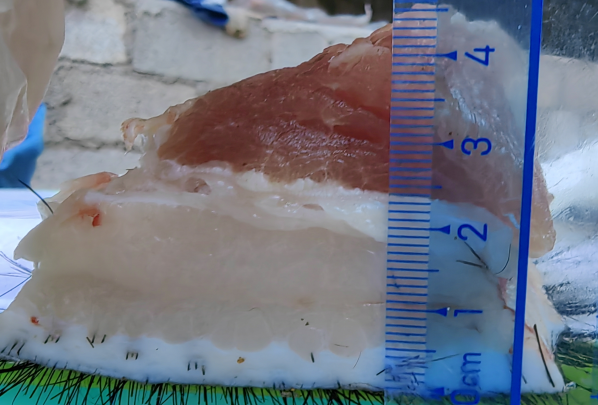

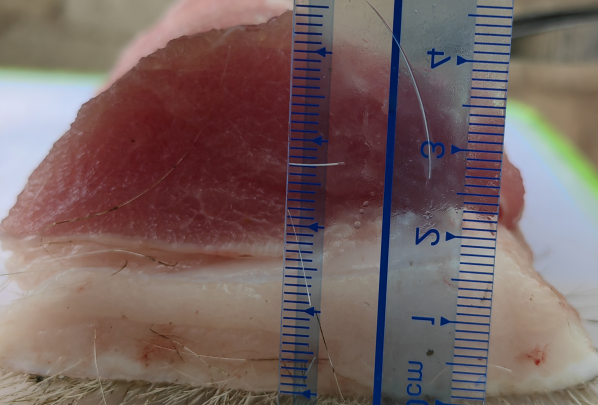


**LW**

**XP**

**Figure S1.** Comparison of backfat thickness between Xiang pigs and Large white pigs.

Supplement: Supplementary file 12 — Supplementary Figure 1. [file 41598_2024_58971_MOESM12_ESM.docx]
